# Enhancing facial nerve regeneration with scaffold-free conduits engineered using dental pulp stem cells and their endogenous, aligned extracellular matrix

**DOI:** 10.1088/1741-2552/ad749d

**Published:** 2024-09-17

**Authors:** Michelle D Drewry, Delin Shi, Matthew T Dailey, Kristi Rothermund, Sara Trbojevic, Alejandro J Almarza, Xinyan T Cui, Fatima N Syed-Picard

**Affiliations:** 1 Department of Bioengineering, Swanson School of Engineering, University of Pittsburgh, Pittsburgh, PA, United States of America; 2 Center for Craniofacial Regeneration, School of Dental Medicine, University of Pittsburgh, Pittsburgh, PA, United States of America; 3 Department of Oral and Maxillofacial Surgery, School of Dental Medicine, University of Pittsburgh, Pittsburgh, PA, United States of America; 4 Department of Oral and Craniofacial Sciences, School of Dental Medicine, University of Pittsburgh, Pittsburgh, PA, United States of America; 5 Center for Neural Basis of Cognition, University of Pittsburgh, Pittsburgh, PA, United States of America; 6 McGowan Institute for Regenerative Medicine, University of Pittsburgh, Pittsburgh, PA, United States of America

**Keywords:** scaffold-free, tissue engineering, peripheral nerve, dental pulp stem cells, neurotrophic factors, nerve regeneration, differentiation

## Abstract

*Objective*. Engineered nerve conduits must simultaneously enhance axon regeneration and orient axon extension to effectively restore function of severely injured peripheral nerves. The dental pulp contains a population of stem/progenitor cells that endogenously express neurotrophic factors (NTFs), growth factors known to induce axon repair. We have previously generated scaffold-free dental pulp stem/progenitor cell (DPSC) sheets comprising an aligned extracellular matrix (ECM). Through the intrinsic NTF expression of DPSCs and the topography of the aligned ECM, these sheets both induce and guide axon regeneration. Here, the capacity of bioactive conduits generated using these aligned DPSC sheets to restore function in critical-sized nerve injuries in rodents was evaluated. *Approach*. Scaffold-free nerve conduits were formed by culturing DPSCs on a substrate with aligned microgrooves, inducing the cells to align and deposit an aligned ECM. The sheets were then detached from the substrate and assembled into scaffold-free cylindrical tissues. *Main results. In vitro* analyses confirmed that scaffold-free DPSC conduits maintained an aligned ECM and had uniformly distributed NTF expression. Implanting the aligned DPSC conduits across critical-sized defects in the buccal branch of rat facial nerves resulted in the regeneration of a fascicular nerve-like structure and myelinated axon extension across the injury site. Furthermore, compound muscle action potential and stimulated whisker movement measurements revealed that the DPSC conduit treatment promoted similar functional recovery compared to the clinical standard of care, autografts. Significance. This study demonstrates that scaffold-free aligned DPSC conduits supply trophic and guidance cues, key design elements needed to successfully promote and orient axon regeneration. Consequently, these conduits restore function in nerve injuries to similar levels as autograft treatments. These conduits offer a novel bioactive approach to nerve repair capable of improving clinical outcomes and patient quality of life.

## 1. Introduction

Approximately 20 million Americans suffer from peripheral nerve injuries per year, equating to an estimated $150 billion in annual health care expenses [1]. Facial nerve injuries, often caused by trauma or surgery, account for a subset of these annual injuries and result in impaired movement and facial paralysis [2]. Since facial movement plays a critical role in communication and social interactions, such injuries have both physiological and psychological impacts, and patients with facial neuromotor disorders are 3–5 time more likely to report depressive symptoms compared to the general population [3, 4]. The most severe facial nerve injuries are characterized by a loss of nerve tissue. The gold-standard treatment for such segmental nerve defects are autografts, which provide a biocompatible option for bridging the nerve gap [5]. This therapy is restricted, though, by tissue availability, loss of function at the donor site, impaired vascularization, nerve ending mismatch after implantation, and risk of infection and neuroma development [6–8].

Even with autograft treatment, though, regeneration remains slow and inefficient, and aberrant reinnervation leads to poor functional outcomes such as synkinesis, defined by abnormally associated muscle movements [3, 9]. Only about 40%–50% of patients receive functional benefits from treatment; thus, many patients with facial nerve injury continue to experience an asymmetric smile, oral incompetence, or lagophthalmos with a potential for corneal injury [6, 10]. To address these deficiencies, nerve guide conduits have been proposed as an alternative method of bridging nerve injuries with volumetric tissue loss. Engineered conduits currently approved for clinical use are hollow tubular constructs composed of either collagen I or synthetic polymers and fail to perform as well as autografts, especially for more severe nerve injuries [5, 11]. Conduits capable of both enhancing axon regeneration and directing axon outgrowth are needed to improve clinical outcomes of facial nerve injuries.

With their innate role in nerve growth and survival, neurotrophic factors (NTFs) are an effective means for promoting axon regeneration. NTFs are growth factors secreted by Schwann cells (SCs), glial cells of the peripheral nervous system, to promote axon regeneration after nerve damage [12]. Exogenous delivery of NTFs, such as brain-derived neurotrophic factor (BDNF) and glial cell line-derived neurotrophic factor (GDNF), has been shown to enhance nerve regeneration in pre-clinical animal models [6, 7, 13]. With their relatively short half-lives and the inhibitory effects of bolus doses, a continuous delivery of NTFs is needed for this therapeutic effect [6, 14, 15]. Cell-based therapies offer a promising alternative for sustained NTF delivery [6]. While SCs would be an ideal candidate for such cell therapies, with their established role in peripheral nerve regeneration, these cells are difficult to isolate and maintain in culture [16].

Alternatively, dental pulp stem/progenitor cells (DPSCs) also intrinsically express high levels of NTFs [17]. Dental pulp is the soft, innervated tissue within the tooth that contains a population of stem/progenitor cells. Like SCs, DPSCs are embryonically-derived from the neural crest, likely explaining their high NTF expression compared to other mesenchymal stem cells [15, 17–19]. These stem/progenitor cells have been previously shown capable of promoting nerve regeneration, both *in vitro* and in pre-clinical animal models [15, 17, 20–30]. Furthermore, DPSCs can be easily isolated from adult third molars and are more immunosuppressive than other mesenchymal stem cells populations; these features make DPSCs a promising and easily accessible source of human autologous or allogenic stem cells, as demonstrated in clinical trials for non-neural applications [23, 31–37]. Our lab has previously shown that DPSCs can form robust cell sheets using scaffold-free tissue engineering techniques [30]. Here, DPSCs become embedded within their endogenous extracellular matrix (ECM), and these tissues act as a cell carrier vehicle for sustained NTF delivery at sites of nerve injury. Our prior work confirmed that DPSC sheets stimulated enhanced nerve regeneration and improved functional outcomes in rat facial nerve crush injury [30].

In addition to providing neurotrophic cues to regenerating axons, DPSC sheets can be engineered to also supply guidance cues to orient axonal outgrowth. Such directional signals are necessary for reducing axon misdirection and thus supporting proper reinnervation and improved clinical outcomes. It has been previously established that axons orient along ECM-scaled topographies, and nerve conduits designed to contain linearly aligned topographies can chaperone regenerating axons towards more accurate reinnervation, consequently improving nerve repair and functional recovery [38, 39]. Similarly, linearly aligned substrate topographies can also induce stem cells to align, elongate, and directionally reorient their autologous ECM [40, 41]. By culturing DPSCs on linear microgrooves, we have previously demonstrated that DPSCs align and deposit a linearly aligned ECM, forming neurotrophic cell sheets capable of inducing linear neurite outgrowth *in vitro* [42].

**Figure 1.**
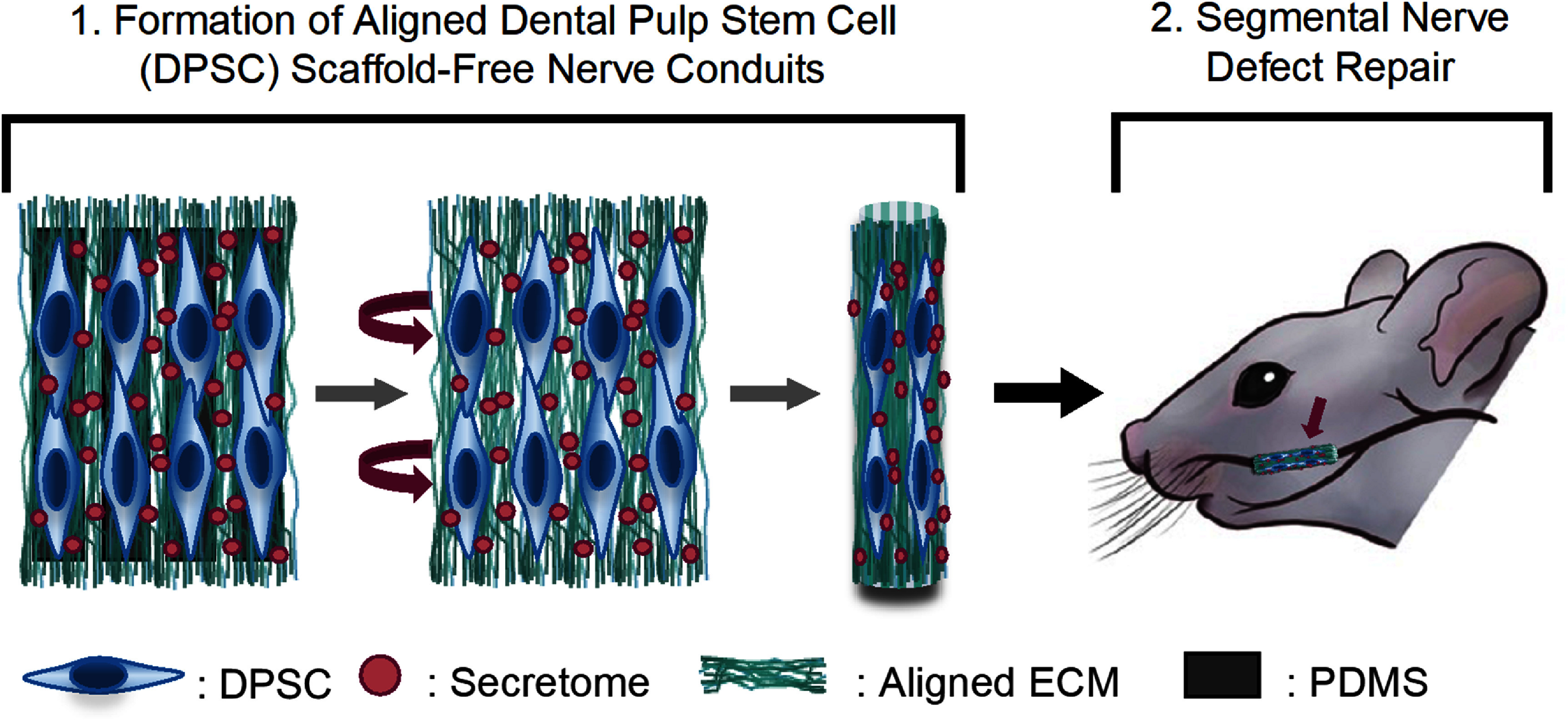
Schematic representation of the formation and implantation of scaffold-free DPSC conduits. Scaffold-free nerve conduits were engineered using DPSCs and their endogenous, aligned ECM. These constructs were then used to bridge a rat segmental nerve defect. DPSC: dental pulp stem cell, ECM: extracellular matrix, PDMS: polydimethyl sulfoxide.

Aligned DPSC sheets provide a promising biomaterial for treating peripheral nerve injuries by providing trophic support to promote axon regeneration and guidance cues to orient axon extension. In this study, we build upon our previous work by assembling our linearly aligned DPSC sheets into cylindrical scaffold-free nerve conduits capable of bridging segmental nerve injuries (figure 1). The aligned DPSC conduit structure, mechanical properties, and NTF expression were evaluated and compared to conduits formed using unaligned DPSC sheets. Furthermore, the capacity of these conduits to restore function in a rodent pre-clinical facial nerve injury model was assessed.

## 2. Materials and methods

### 2.1. Isolation of dental pulp stem cells

Healthy human third molars from the University of Pittsburgh School of Dental Medicine were used to isolate dental pulp stem/progenitor cells (DPSCs), as previously documented [30, 42–44]. Briefly, teeth were collected and stored in 1X phosphate buffered saline (PBS; Gibco) with 1X penicillin and streptomycin (P/S; Gibco), and within 24 h post-extraction, the isolated pulp was minced and enzymatically digested at 37 °C for 1 h using 3 mg ml^−1^ collagenase (EMD Millipore) and 4 mg ml^−1^ dispase (Worthington Biochemical). The resulting DPSCs were filtered using a 70 *μ*m cell strainer and then cultured with Dulbecco’s Modified Eagle Medium (DMEM; Gibco), 20% fetal bovine serum (FBS; Atlanta Biologicals), and P/S, passaging and cryopreserving the cells at 80% confluence. Cryopreservation was accomplished in a DMEM, 20% FBS, 1X P/S and 10% dimethyl sulfoxide (DMSO; Fisher). Only DPSCs from passage 2-5 were used to form conduits.

### 2.2. DPSC conduit formation

DPSCs were cultured on either a flat substrate or a substrate with linear microgrooves, as described previously [42]. These linearly grooved substrates were formed using Sylgard 184 polydimethylsiloxane (PDMS) cured on a silicon wafer with negative features, forming an array of parallel pillars 10 *μ*m wide, 5 *μ*m deep, and spaced 10 *μ*m apart. The flat polymer substrates were made by curing PDMS on a flat surface. All molds were cut to fit within 6-well plate wells, then coated with 2 *μ*g cm^−2^ laminin (Gibco), and UV sterilized. The DPSCs were cultured on the laminin-coated PDMS substrates in a growth media containing DMEM, 20% FBS, 1% P/S, 50 *µ*g ml^−1^ L-ascorbic acid (Sigma-Aldrich), and 5 ng ml^−1^ fibroblast growth factor (FGF; Peprotech) at a cell density of approximately 42 000 cells cm^−2^ [30, 42]. The culture media was changed every 2–3 d until the DPSCs formed a robust cell sheet. At this point, the tissues were rolled up to form cylindrical constructs and transferred to flat, UV sterilized PDMS. Stainless steel minutien pins (Fine Science Tools) were placed on either end of the constructs, 7 mm apart, securing the constructs to the PDMS. After 2 d, the rolled-up sheets fused to form solid cylindrical tissues, thus manufacturing aligned conduits from the DPSC sheets cultured on the microgroove substrate and unaligned conduits from DPSC sheets form on the flat substrate.

### 2.3. Geometric analysis of DPSC conduits

The cross-sectional areas of the scaffold-free DPSC conduits were quantified by measuring the diameter of each conduit at 3 locations and using an average diameter to calculate the cross-sectional area. This was repeated for biological replicates from aligned conduits (*n* = 4), unaligned conduits (*n* = 4), and rat buccal branch nerves (*n* = 3).

### 2.4. Histological analysis of the DPSC conduits

The DPSC conduits were fixed in 10% formalin after washing twice in PBS. The conduits then underwent standard processing for paraffin embedding and were cut into 5 *µ*m sections. Sections were stained using hematoxylin and eosin (H&E) or picrosirius red. Immunofluorescent staining was done with primary antibodies against BDNF (ThermoFisher; raised in rabbit and used at a dilution of 1:250) or GDNF (ThermoFisher; raised in rabbit and used at dilution of 1:250) and secondary antibody Alexa Fluor 488 (ThermoFisher; goat anti-rabbit and used at a dilution of 1:500). DAPI was used to stain the nuclei. The Nikon ECLIPSE Ti, ZEISS Scope.A1 AXIO or Nikon TE 2000 microscopes were used to capture brightfield and fluorescent images, and ImageJ was used to process the images.

### 2.5. Scanning electron microscopy (SEM)

The DPSC conduits were fixed in 2.5% glutaraldehyde (Sigma-Aldrich) and post-fixed with osmium tetroxide (Sigma-Aldrich). The conduits were then dehydrated through a series of ethanol washes. After drying, the tissues were mounted on a metal stub and sputter-coated using gold. A JSM 6335F scanning electron microscope (Top Analytica) was used for imaging, and the resulting images were processed using the ImageJ software.

### 2.6. Enzyme-linked immunosorbent assay (ELISA)

To quantify the expression of NTFs within the DPSC conduits, fresh tissues were flash frozen in liquid nitrogen and pulverized using a pestle. A solution of RIPA buffer/Triton X-100 (Thermo Fisher) and 50X protease inhibitor cocktail (Promega) was used to generate a protein lysate from the aligned and unaligned DPSC conduits. Then human BDNF (PicoKine ELISA Kit, Boster Biological Technology) and GDNF (PicoKine ELISA Kit, Boster Biological Technology) ELISA kits were used to measure the NTF concentrations in the lysates. These assays were performed based on manufacturer’s instructions, extrapolating the protein concentrations from standard curves quantified with manufacturer-provided standards. Figures were generated using biological replicates (*n* = 3), and statistical analysis was done with paired t-tests to detect difference in NTF levels between unaligned and aligned conduits.

### 2.7. Tensile tests

For tensile testing, the ends of the tissues were glued between two strips of 400 grit sandpaper to prevent slipping and then placed into a dish of PBS to remove bubbles formed within the strips of sandpaper. An Insight 1 kN testing apparatus with a 10 N load cell (MTS) was used to perform tensile testing within an attached container filled with 1X PBS. All testing was done at room temperature. The samples were gripped between customized clamps and preloaded to 0.01 N at a constant rate of 2 mm min^−1^, and sample length after preload was recorded. The samples were then pulled until failure at this same rate of 2 mm min^−1^. Time, load, and displacement were recorded at an acquisition rate of 100 Hz by the MTS Testworks 4 software. Peak load, total elongation, and all raw force-displacement data were collected. This was done for biological replicates of the aligned (*n* = 4) and unaligned (*n* = 4) conduits and rat facial nerves taken from the buccal branch (*n* = 3); averages were taken from 2 technical replicates for each biological replicate. For each sample, the ultimate load (N), peak load (N), percent displacement at failure (mm), and stiffness (N mm^−1^) were calculated. Failure was defined as a 10% drop in the measured load that persisted for at least 10% elongation of the original height. Statistical analyses were performed using one-way analysis of variance (ANOVA) and Tukey’s post-hoc tests, and figures were created using RStudio [45].

### 2.8. Implantation of DPSC conduits in rat facial nerve defect

All animal procedures were approved by the University of Pittsburgh Institutional Animal Care and Use Committee (Protocol No. 21038670). One day prior to surgery, Sprague Dawley rats were switched to a diet supplemented with cyclosporine A (100 pm; Envigo) to induce immunosuppression and were kept on this diet for the duration of the experiment. During surgery, the rats were anesthetized with an intraperitoneal injection of 40 mg kg^−1^ ketamine and 5 mg kg^−1^ xylazine. The facial nerve was exposed through a preauricular incision in the rat face, and 5 mm of the buccal branch was removed. For the conduit experimental group (*n* = 13), an aligned DPSC conduit was sutured to the host nerve with two single knots of a 9-0 nylon monofilament suture at each of the nerve ends. Conduits generated from DPSCs isolated from two separate human patients were utilized in these experiments. In the autograft group (*n* = 10), the explanted tissue was rotated 180° and sutured similarly into the nerve gap. As for the negative control group (*n* = 3), the nerve gap was left empty, and the nerve ends were sutured to surrounding facia. For all groups, a 5-0 resorbable gut suture was used to close the wound. Based on initial *in vitro* analyses, only aligned conduits, but not unaligned conduits, were capable of providing both NTF support and an aligned topography to promote and orient axon extension; therefore, only aligned conduits were utilized in these *in vivo* experiments.

### 2.9. CMAP and whisker movement analyses

Twelve weeks after surgery, the animals were anesthetized with inhalation of 3% isoflurane in oxygen and maintained at 2%–2.5%. Similar to previously described, surgical regions were shaved and cleaned with ethanol, and a stainless-steel bone screw (Fine Science Tools) was placed on the skull for use as a reference electrode [30]. The buccal branch was exposed and a micro-cuff electrode (Microprobes for life science, NC-1-4-100 *µ*m Pt-1-1-SUT-300 *µ*m-CON) was placed on the proximal end of the nerve as a bipolar stimulation electrode. Additionally, the marginal mandibular branch of the facial nerve was transected to ensure that recordings only captured signals propagated by the stimulated buccal branch. The rat’s head was then fixed with ear bars on a stereotaxic frame. Charge-balanced biphasic stimulation was delivered through the cuff electrode and the nerve was stimulated using the following parameters: 0–1500 *µ*A, 1 Hz for 20 s, biphasic symmetric pulse with cathodic leading, 100 *µ*S each phase, and 100 *µ*S interphase delay. The nerve was kept hydrated with sterile PBS throughout the surgery and recording session. Stimulation and recording experiments were conducted using the Ripple Grapevine processor stim/record system (Nano2 + Stim headstage). Evoked EMG activities were recorded with an in-house fabricated multistrand stainless steel wire electrode (Cooner wire AS632) inserted underneath the whisker pad with a hypodermic needle. To evaluate the regenerated neuromuscular functions, the nerve was stimulated with different currents ranging from 0 *µ*A to supramaximal 1500 *µ*A. The resulting compound muscle action potential (CMAP) data was processed with MATLAB (MathWorks). At least 10 individual CMAP responses were averaged to produce the mean CMAP waveform for each stimulation current, and peak-to-peak amplitude was calculated as the difference between the lowest negative peak and highest positive peak. Area under the curve (AUC) was calculated as the area between the CMAP traces and the *x* axis within a 6 ms time window after the stimulation onset. The area calculation utilized the MATLAB trapezoidal numerical integration method to compute the approximate integral of voltage values (assume unit spacing). The time window was chosen to ease the calculation because most of the observed CMAP responses fade away 6 ms after stimulation. Furthermore, a sigmoid function was used to fit the muscle recruitment curve [46] (supplemental figure 1 and supplemental table 1) using the following equation:

\begin{align*}y = f\left( x \right) = c\frac{c}{{1 + {e^{ - b\left( {x - a} \right)}}}}\end{align*}

where *a* is the logistic sigmoid’s midpoint, *b* is the logistic growth rate, and *c* is the logistic maximum amplitude. After fitting a pre-established function to the curve, the activation threshold was defined as 20% the maximum plateau amplitude [47]. Correlation analysis between stimulation strength and muscle response was also performed in Matlab using the Matlab corr function (supplementary table 2). Electrophysiological data was collected for the conduit treated (*n* = 7), autograft treated (*n* = 6), and uninjured (*n* = 15) nerves. At the end point of the studies, empty nerve defects lacked any regenerated nerve tissue across the injury site therefore these animals were not evaluated.

Evoked whisker movement was recorded with a digital camera (iPhone 11, 1920 × 1080 resolution, 240 frames per second) for quantitative processing. The camera was positioned above the whisking plane of the head-fixed rat and a dark pad was placed underneath the whisker as background for better contrast. During video recording, the nerve was stimulated with either 0 *µ*A current as negative control or supramaximal 1500 *µ*A current to evoke maximum whisker movement. Videos were cropped in a video editing software (Kdenlive), zooming in on the whiskers and exported to image sequences for ImageJ quantification. Whiskers from the third column were used for analysis. Frames of maximal retraction and maximal protraction were manually identified, and the angular difference was measured using ImageJ. Whisker function was also measured for the conduit treated (*n* = 7), autograft treated (*n* = 6), and uninjured (*n* = 8) nerves. Statistical analyses for both CMAP and whisker movement were performed in RStudio or GraphPad Prism using the Kruskal–Wallis test and the Mann–Whitney–Wilcoxon test [45].

### 2.10. Histological analysis of the nerve explants

Immediately after CMAP and whisker movement analyses, the rats were euthanized, and the buccal branch of the facial nerve was explanted and fixed in formalin. The tissues were then embedded in Tissue Plus O.C.T Compound (Fisher Scientific), frozen in liquid nitrogen-chilled isopentane, and cryo-sectioned at a thickness of 5 *μ*m. Sections were stained using H&E. Immunofluorescent staining was also performed using primary antibodies against Neurofilament-M (BioLegend; raised in rabbit and used at a 1:100 dilution), growth-associated protein 43 (GAP43) (Abcam; raised in rabbit and used at a dilution of 1:50), and myelin basic protein (MBP) (Proteintech; raised in rabbit and used at 1:250) and secondary antibody Alexa Fluor 546 anti-rabbit IgG used at a dilution of 1:500 (ThermoFisher); DAPI was also included to stain nuclei. The Nikon ECLIPSE Ti, ZEISS Scope.A1 AXIO or Nikon TE 2000 microscopes were used for imaging these stains, and the images were processed using ImageJ. Quantitative image analysis was performed on the MBP stained images with ImageJ, using the area selection tool and the analyze particles function to measure axon area. The number and area of all discernable axons within 3 fields of views per each location along the explant were determined, and one biological sample per group was assessed. The axon area and number of myelinated axons were then compared between the uninjured, autograft, and conduit-treated explants for each location. Since the empty defects lacked any regenerated nerve tissue, this experimental group was omitted from these analyses. RStudio was used to create the figures and perform statistical analyses [45]. For the axon area data, the Kruskal–Wallis test and the Mann–Whitney–Wilcoxon test were used for statistical analyses, whereas ANOVA and Tukey’s post-hoc tests were used to evaluate the axon count data.

## 3. Results

### 3.1. Structural and histological characterization of scaffold-free DPSC conduits

**Figure 2.**
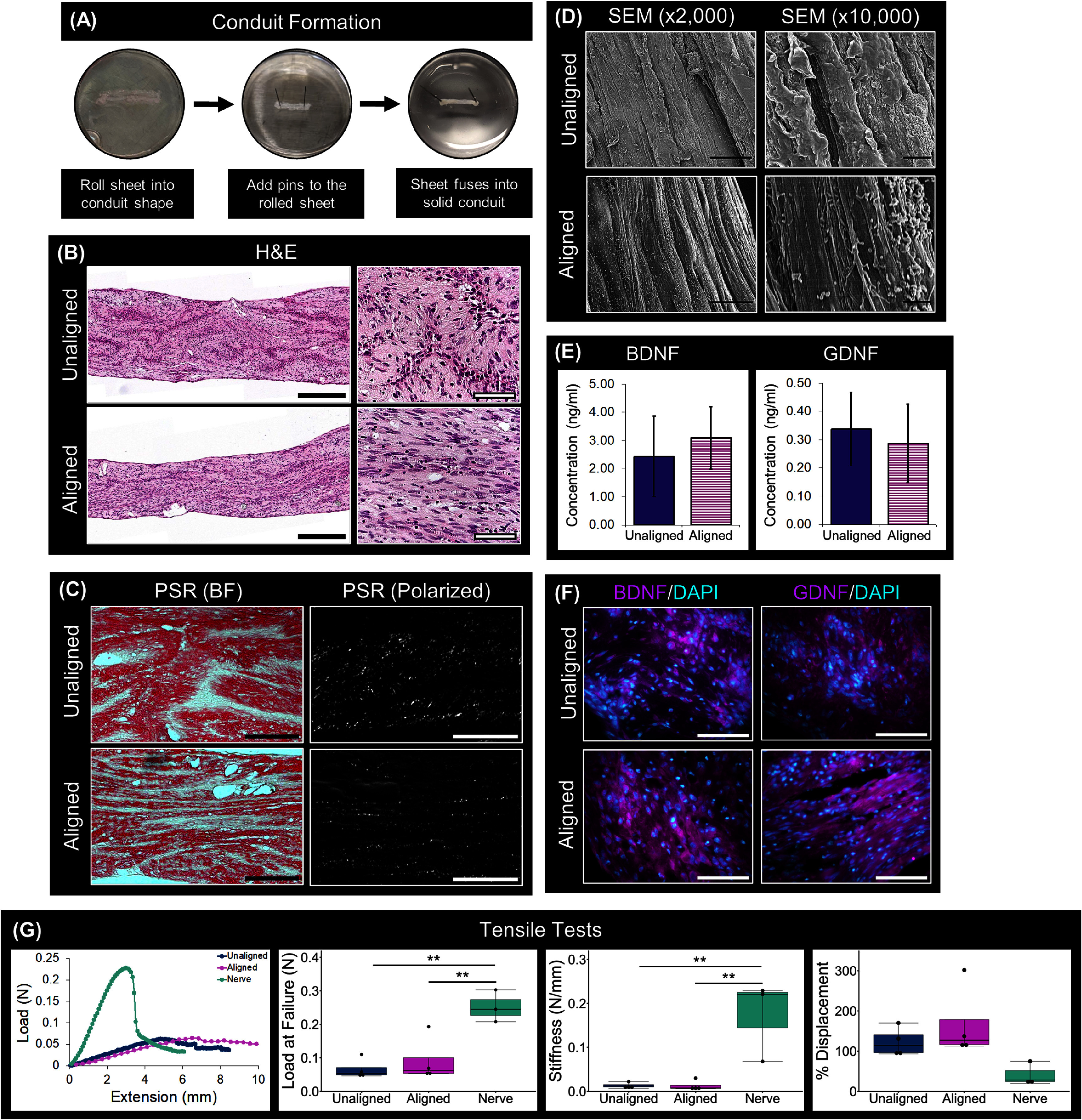
Formation and characterization of aligned DPSC conduits. (A) Images of conduit formation. (B) Hematoxylin and eosin (H&E) staining of longitudinal dental pulp stem cell (DPSC) conduit sections showed that both unaligned and aligned conduits were solid and cellular, with those formed from the aligned DPSC sheets maintaining their nuclear alignment. (C) Picrosirius staining (PSR), imaged with brightfield (BF) and polarized light microscopy, indicated that the conduits from the aligned DPSC sheets also preserved their extracellular matrix (ECM) alignment. (D) Scanning electron microscopy (SEM) further confirmed that the aligned DPSC conduits contained an oriented matrix which was absent from the unaligned conduits. (E) Neurotrophic factor (NTF) expression, quantified using ELISA, indicated similar levels of brain-derived neurotrophic factor) and glial cell line derived neurotrophic factor (GDNF) in the conduits. Paired t-test indicated that the NTF expression levels were statistically similar between the unaligned and aligned conduits. (F) Immunofluorescence staining against BDNF and GDNF demonstrated that this NTF expression was present throughout the matrix of the conduits. (G) Tensile tests produced load-displacement curves such as those represented here. From these curves the load at failure (N), stiffness (N mm^−1^), and % displacement were calculated, overall showing that these factors for the conduits were on the same order of magnitude as rat facial nerves. Statistical comparisons for the mechanical assessments were performed using one-way analysis of variance (ANOVA) and Tukey’s post-hoc tests (*: *p*-value < 0.05, **: *p*-value < 0.01). Scale bars: (B) low magnification = 500 *μ*m, high magnification = 200 *μ*m, (C) BF = 150 *μ*m, polarized = 150 *μ*m, (D) ×2000 = 15 *μ*m, ×10 000 = 2 *μ*m, (F) 100 *μ*m.

Scaffold-free conduits were engineered by first culturing DPSCs on either a flat or microgrooved substrate, as previously described [42], forming un-aligned and aligned DPSC sheets, respectively. These cell sheets were then assembled into a cylindrical shape and subsequently remodeled to fuse forming solid scaffold-free tissues (figure 2(A)). The aligned conduits had an average cross-sectional area of 0.92 ± 0.38 mm^2^ and the unaligned conduits an average of 1.50 ± 0.92 mm^2^. The capacity of these conduits to restore function in a rat facial nerve injury was subsequently evaluated; importantly, the cross-sectional areas of the engineered conduits were statistically similar to that of the buccal branch of the rat facial nerve (0.60 ± 0.18 mm^2^).

Previously, we had established that culturing DPSC sheets on grooved substrates induced the DPSCs to align and subsequently deposit an aligned collagenous ECM capable of orienting axon extension [42]. The capacity of conduits generated using these respective cells sheets to maintain this initial cell and ECM alignment was now evaluated. H&E staining of longitudinal sections of the conduits showed that both the aligned and unaligned conduits were robust and cellular. Furthermore, the DPSC nuclei were linearly aligned in the conduits generated using the aligned cell sheets. (figure 2(B)). Picrosirius red staining visualized using polarized light microscopy verified that the linearly aligned DPSC sheets preserved their collagenous ECM alignment upon conduit formation (figure 2(C)). SEM further validated this matrix configuration, showing that at higher magnifications the conduits formed from the aligned sheets contained a linearly oriented matrix that was not present in the conduits from the unaligned sheets (figure 2(D)). These data demonstrate that DPSC and ECM alignment established during cell sheet formation is maintained after the sheets have coalesced into cylindrical tissues suggesting that aligned DPSC conduits will provide a conducive substate for guiding regenerating axon extension.

### 3.2. NTF expression by scaffold-free DPSC conduits

The capacity of DPSC-based conduits to produce trophic cues to promote axon regeneration was evaluated. ELISA confirmed that the DPSC conduits endogenously expressed the NTF proteins BDNF and GDNF (figure 2(E)) and this expression was statistically similar between the aligned and unaligned conduits. NTF localization was further assessed by immunostaining on longitudinal sections of the conduits, which demonstrated BDNF and GDNF expression was evenly dispersed throughout the matrix of both types of conduits (figure 2(F)).

### 3.3. Mechanical properties of scaffold-free DPSC conduits

Ideally, nerve conduits should have comparable mechanical properties to the native nerve. Tensile tests were performed to evaluate the mechanical properties of the scaffold-free conduits (figure 2(G)). The native rat nerve tissues were significantly stiffer and allowed greater load at failure than the aligned (*p* = 0.048 for stiffness and 0.0087 load at failure) and unaligned (*p* = 0.048 for stiffness and 0.0036 load at failure) conduits; these mechanical parameters were statistically similar between the unaligned and aligned conduits. In contrast, the percent displacement at failure was similar between the conduits and native rat facial nerves. Although the native nerve tissue is stiffer and can withstand a greater load at failure, the conduits exhibit values for these parameters that are within the same order of magnitude as the nerve.

### 3.4. Histological analyses of rat facial nerves treated with aligned scaffold-free conduits

**Figure 3.**
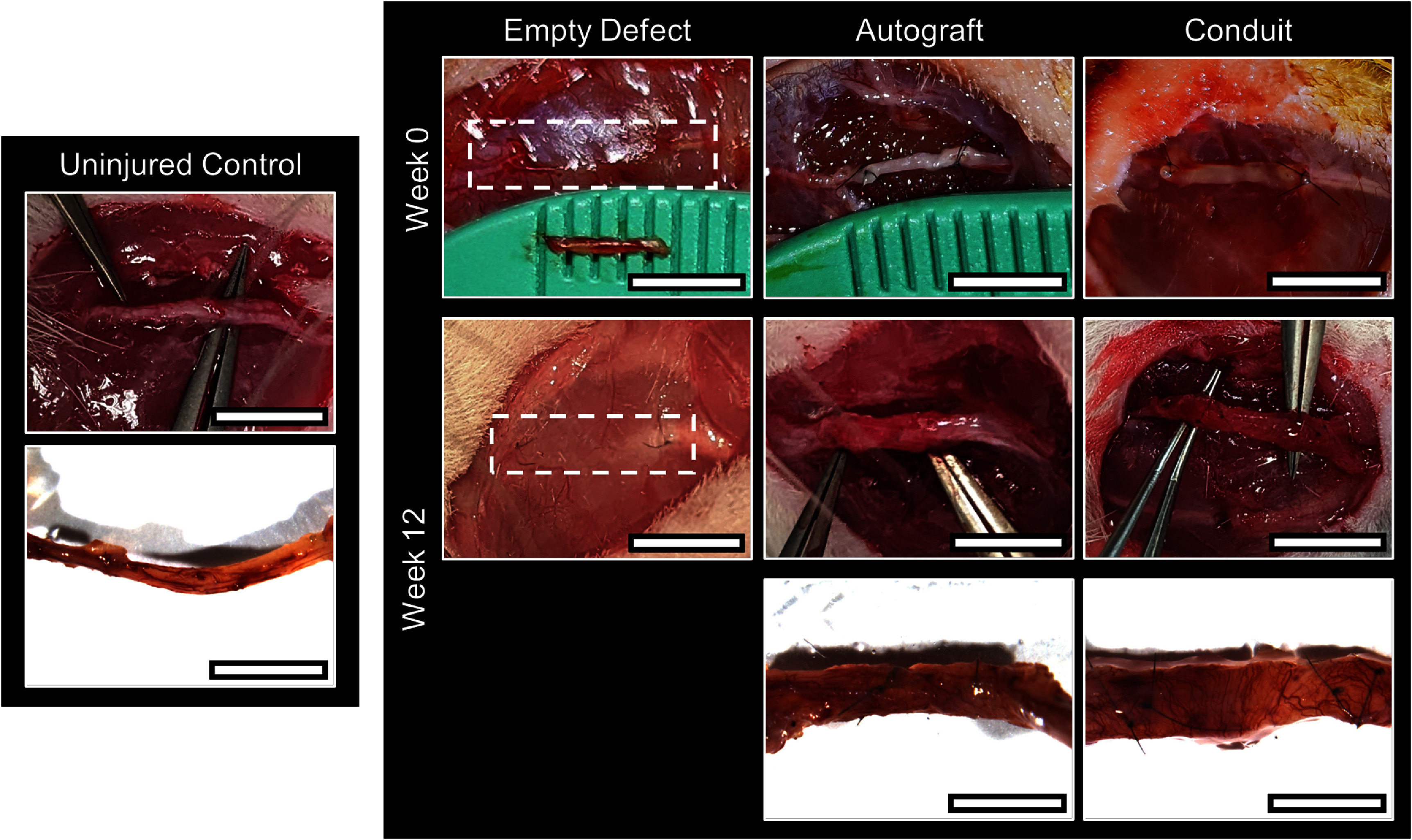
Images of the segmental nerve defect and treatments at week 0 and week 12. Nerve tissue from the autograft and conduit treatments were explanted and compared to uninjured controls. Scale bars: Week 0: = 4 mm, Week 12 = 5 mm, Explants = 3 mm.

The capacity of aligned DPSC conduits to promote axon regeneration and restore nerve function *in vivo* was evaluated in critical-sized segmental injuries of the buccal branch of the rat facial nerve. Since the *in vitro* studies confirmed that the aligned DPSC conduits had similar NTF expression levels as unaligned conduits to promote axon extension and provided the added property of an aligned ECM to orient axon extension, only the aligned DPSC conduits were further evaluated in these *in vivo* studies. Three experimental conditions were evaluated: nerves were either bridged with a linearly-aligned DPSC conduit, treated with an autograft to emulate the current standard of care, or the injury site was left empty as an untreated negative control. After 12 weeks, tissue regrowth was not observed in the untreated nerves, yet robust tissue was bridging the injury site in the nerves treated with the autografts and conduits (figure 3).

**Figure 4.**
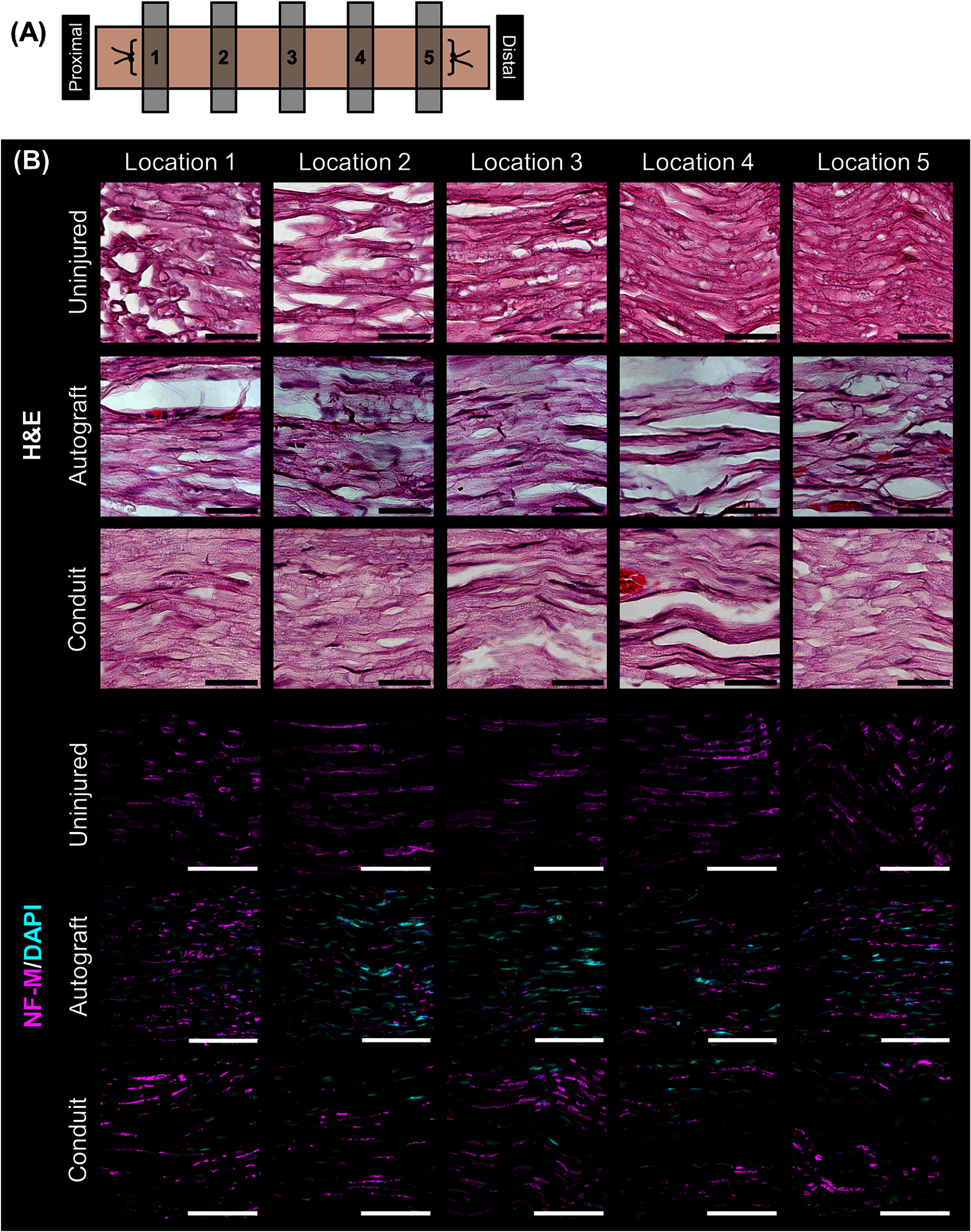
Histological analysis of longitudinal explant sections. (A) Schematic depicting locations along the explant where images were taken. (B) Hematoxylin and eosin (H&E) staining showed solid tissue throughout the regenerated regions of the explants that looked like the nerve tissue of the uninjured control. Immunofluorescence staining against neurofilament-M (NF-M) (magenta) showed axons extending across the explants, with those treated with dental pulp stem cell (DPSC) conduits more closely resembling uninjured nerves. DAPI staining was used to visualize nuclei in fluorescent-stained images (cyan). Scale bars: (B) H&E = 75 *μ*m, NF-M/DAPI = 100 *μ*m.

Histological analysis was performed on longitudinal sections of the explants and images were acquired at 5 locations along the length of the tissue (figure 4(A)). H&E staining revealed that nerves treated with autograft tissue or the aligned DPSC conduits comprised a solid tissue with a structure reminiscent of native nerve tissue (figure 4(B)). Axons extended across the injury site in nerves treated with both autografts and conduits as visualized by immunohistochemical staining against NF-M (figure 4(C)). NF-M expression was less intense and more punctuated in the autograft group compared to the uninjured nerves, especially at the center of the explant. In contrast, the NF-M expression patterns of the conduit group more closely resembled that of uninjured nerves, particularly at the proximal and center of regions of the conduit.

**Figure 5.**
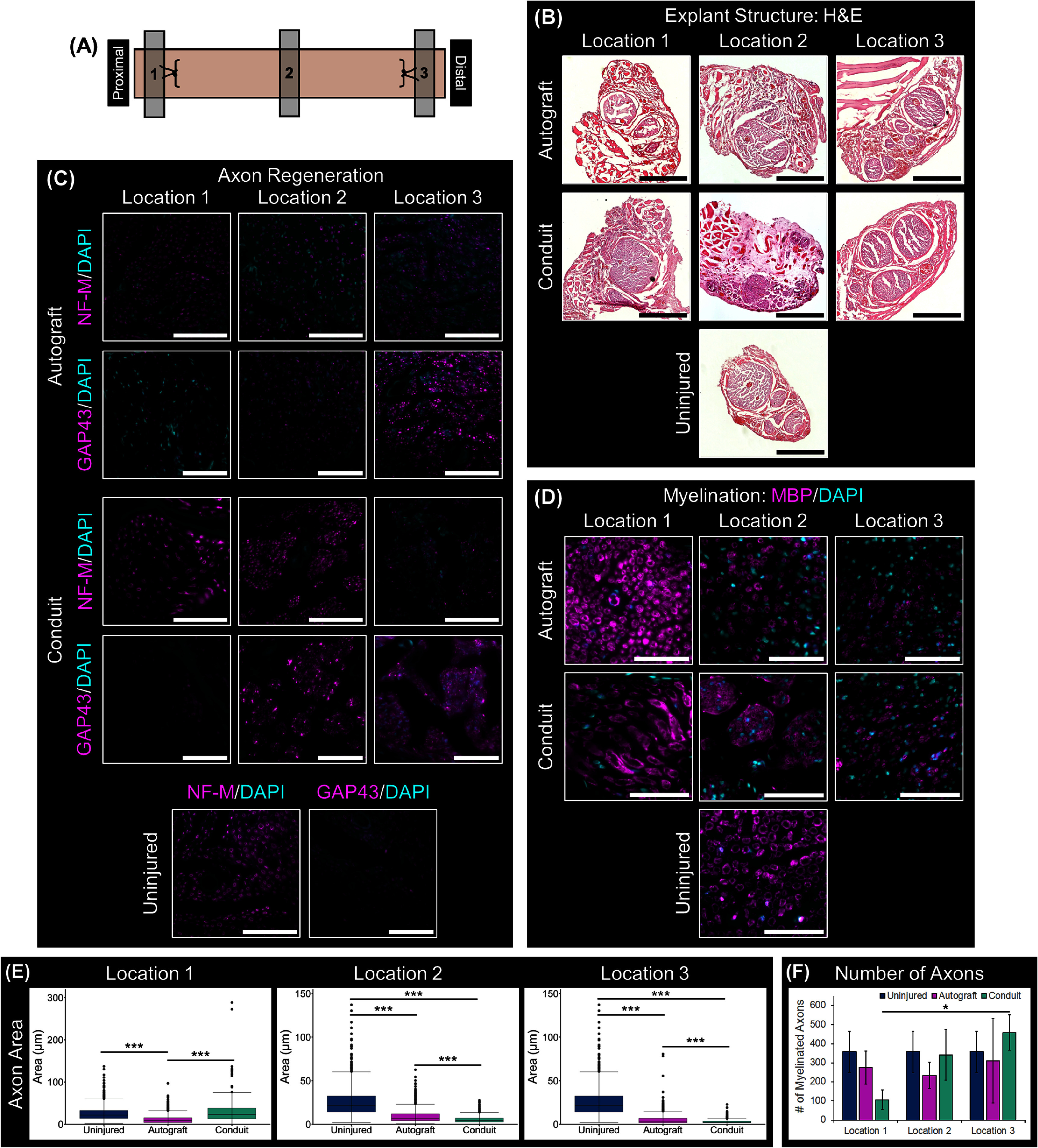
Histological analysis of explant cross-sections. (A) Schematic indicating imaging locations along the tissue explants. (B) Hematoxylin and eosin (H&E) staining confirmed the presence of neural-like tissue within the explants, as well as outside the proximal and distal sutures. (C) Immunostaining against neurofilament-M (NF-M) (magenta) and growth associated protein 43 (GAP43) (magenta) validated the presence of neurons throughout all explants, with increases in GAP43 from the proximal to distal ends being correlated with decreases in NF-M. (D) Immunostaining against myelin basic protein (MBP) (magenta) showed that these axons were myelinated, with decreased myelination at the distal ends of the treated nerves. DAPI staining was used to visualize nuclei in fluorescent-stained images (cyan). (E) The area of myelinated axons varied between treatment group and location. Statistical comparisons were made using Kruskal–Wallis test and the Mann–Whitney–Wilcoxon test (F) Conduit and autograft treatment resulted in a similar number of myelinated axons across the explant compared to the uninjured control. However, an increase in axon density was detected in the conduits from proximal to distal regions of the explant. Statistical comparisons were made using ANOVA and Tukey’s post-hoc tests (*: *p*-value < 0.05, **: *p*-value < 0.01, ***: *p*-value < 0.001). Scale bars: (B) 400 *μ*m, (C) 100 *μ*m, (D) 75 *μ*m.

Histological cross-sections of the explants were taken proximal and distal to the injury site and at approximately the center of treatment region, as demonstrated in figure 5(A). The conduit and autograft explants contained fascicle-like structures that resembled those in the uninjured control nerves, though the size of these fascicles were larger in some regions and smaller in others (figure 5(B)). Conduit-treated nerves consistently exhibited an increased number of smaller fascicles within the center of the explant. H&E staining also demonstrated that explants from both experimental groups were vascularized throughout the length of the tissue.

Immunofluorescent characterization of the nerve cross-sections further confirmed myelinated axon regeneration in both autograft and conduit treated nerves (figures 5(C) and (D)). Neurofilaments, a class of intermediate filaments, is associated with mature axons whereas GAP43 is a protein found in developing and regenerating axons. Correspondingly, strong NF-M expression and minimal GAP43 expression can be detected in healthy, uninjured nerve tissues (figure 5(C)). NF-M signal was more pronounced in the conduit group than in the autograft. Moreover, in both treatment conditions, an inverse correlation was observed between the expression levels of NF-M and GAP43, where a decrease in NF-M expression and increase in GAP43 expression was detected from the proximal to distal ends of the explants suggesting active axon regeneration and maturation across the injury site (figure 5(C)). The axons extending across both autograft and conduit treated nerves were myelinated as demonstrated by positive MBP staining (figure 5(D)). Like NF-M expression, MBP expression was stronger at the proximal end and decreased towards the distal end for both experimental groups.

Quantitative image analyses (figure 5(E)) measuring the individual axon areas showed that, on average, the axons were significantly larger at the proximal end compared to the distal end for both treatment groups (*p* < 0.001). Axons in both autograft and conduit-treated nerves, though, were significantly smaller than those of the uninjured nerve (*p* < 0.001) at the center and distal end of the explants; however at the proximal end, the axons were similar in size between the conduit treated explants and the healthy nerve, but the axons in the autograft treatment group were smaller. In contrast, counting the number of axons showed that axon density was statistically similar among the healthy, autograft and conduit-treated nerves at each location (figure 5(F)). The only exception was the significantly greater number of axons at the distal region of the conduit treated nerve (location 3) compared to the proximal region (location 1) (*p* = 0.025). Overall, histological analyses of the nerve explants demonstrated that treatment of a segmental nerve defect with aligned scaffold-free nerve conduits resulted in regeneration of myelinated axons extending from the proximal to the distal end of the injury.

### 3.5. Evaluation of functional nerve regeneration *in vivo*

**Figure 6.**
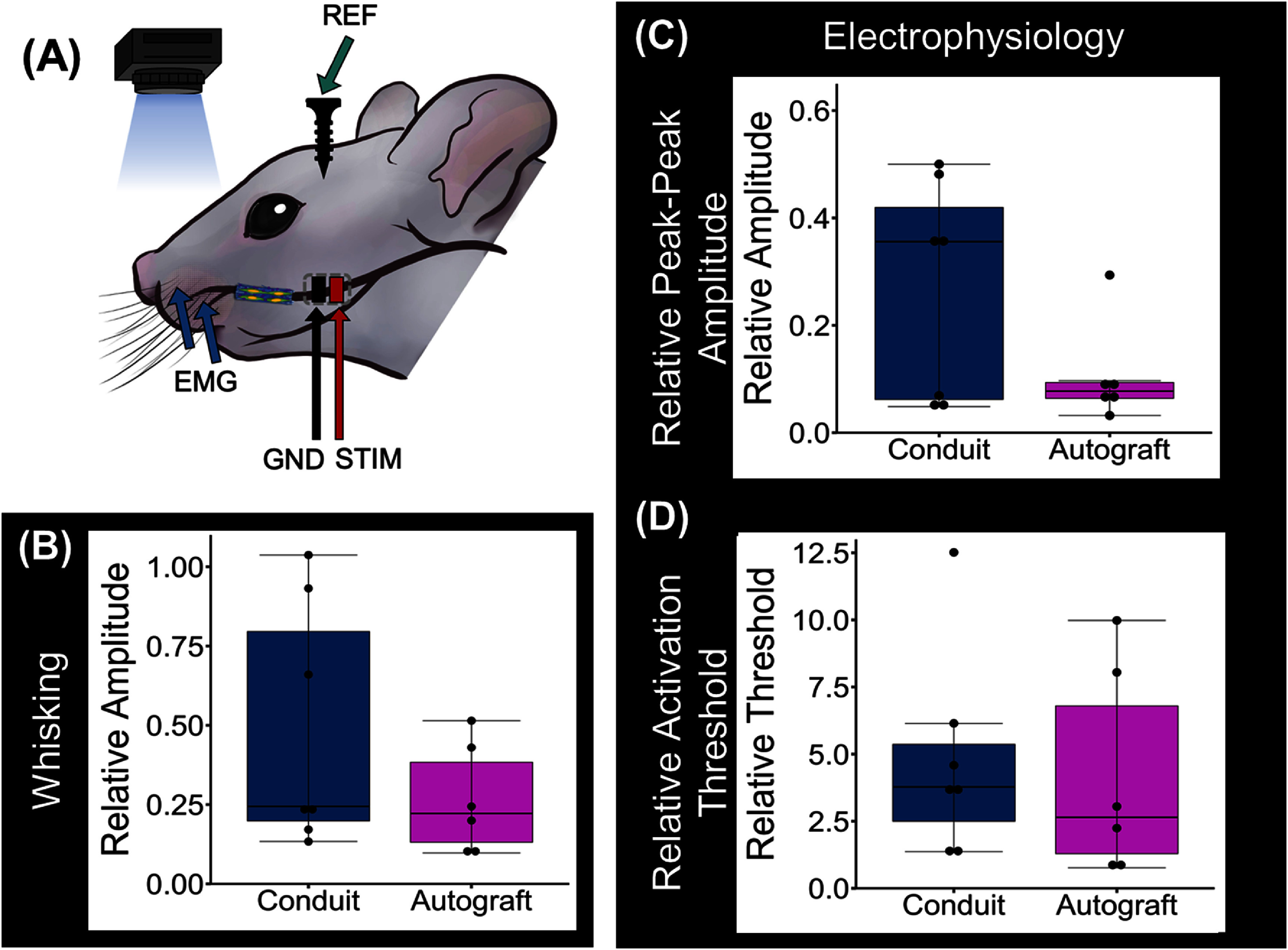
Functional evaluation of nerves treated with DPSC conduits or autografts relative to healthy nerve. (A) Schematic of the set-up used to record compound muscle action potential (CMAP) and whisking data. (B) Whisker motion amplitude was statistically similar between autograft and conduit treated nerves (*p* = 0.32), but there was greater variance in the conduit group where some animals performed as well as uninjured nerves and others more similarly to the autograft-treated nerves. (C) CMAP peak-to-peak amplitude and (D) activation threshold measurements likewise showed statistically similar evoked activity between the autograft and conduit-treated nerves with a wide range of activity in nerves treated with the DPSC conduits. (peak-peak amplitude: *p* = 0.37; activation threshold *p* = 0.53). Whisking and CMAP analyses are presented relative to healthy controls. Statistical analyses for both CMAP and whisker movement were performed in RStudio using the Kruskal–Wallis test and the Mann–Whitney–Wilcoxon test. REF: reference, EMG: electromyography, GND: ground, STIM: stimulation.

Functional recovery was determined by electrically stimulating the treated nerves proximal to the injury site and measuring evoked whisker movement and the action potential within the vibrissal muscle relative to healthy condition (figure 6(A)). The aligned scaffold-free conduits restored whisker motion with the normalized whisking motion amplitude statistically similar to the autografts (figure 6(B)); supplemental figure 2(A)). CMAP measurements further confirmed that the conduit and autograft treated nerves required similar levels of current to reach maximum activation and exhibited similar peak-to-peak amplitude (figures 6(C) and (D), supplemental figures 2(B) and (C), supplemental figure 3(A)). Furthermore, the area under the curve of the CMAP traces was statistically similar (*p* = 0.1807) between the autograft and conduit treated groups (supplemental figures 3(B)–(D)). These data confirm that scaffold-free aligned DPSC-based conduits can restore the function of injured rat nerves to levels comparable to autograft treatments.

## 4. Discussion

Challenges exist in engineering synthetic nerve conduits that can provide equivalent outcomes as autograft tissues in the treatment of severe nerve injury. Here, we establish that scaffold-free conduits comprising DPSCs and their endogenous, aligned ECM facilitate the regeneration of a vascularized fascicular nerve-like structure when implanted across critical-sized defects in the rat facial nerve. These bioactive conduits promote the extension of myelinated axons across the injury site and restore nerve function to similar levels as autograft controls. Scaffold-free tissue engineering offers several benefits over traditional scaffold-based designs, such as improving the incorporation and distribution of cells throughout the engineered construct, and providing a biomimetic microenvironment that preserves natural cell–cell and cell–ECM interactions [48]. Moreover, scaffold-free approaches avoid the use of exogenous scaffold materials that are known to strongly affect cell phenotype and can adversely affect healing. Prior attempts of engineering scaffold-free conduits for peripheral nerve repair utilized different techniques such as 3D bioprinting, which is costly and often puts undue stress on the cells, and none contained DPSCs or an aligned substrate topography [49–54], which importantly provide the neurotrophic support and guidance cues needed effectively promote and orient axon extension.

Even with treatment, peripheral nerve repair often takes 19 months yet the regenerative capacity of nerves begins to decline after 1–2 months of denervation [55]. At this point, there is progressive loss of Schwann cell support and subsequent Schwann cell senescence; this results in decreased expression of NTF and basal lamina-associated ECM molecules, increased collagen remodeling, and changes in bands of Büngner architecture [6, 7, 12–14, 56–58]. When assembled into nerve conduits, aligned DPSC sheets preserved their linearly oriented ECM and intrinsic NTF expression emulating these important elements of natural nerve repair processes. We have previously shown that NTF production levels of DPSC sheets are sufficient to enhance axon regeneration in a nerve crush injury animal model, and, when in direct co-culture, linearly aligned DPSC sheets promote oriented neurite extension in neuronal cells *in vitro* [30, 42]. Therefore, when implanted in an *in vivo* nerve injury site, these critical features of the aligned DPSC conduits likely supplemented the innate nerve repair mechanisms that are diminished with chronic denervation and consequently promoted repair.

Histological analyses demonstrated that the aligned DPSC conduits induced the regeneration of myelinated axons across the segmental nerve defect. The size of these fibers decreased from the proximal to the distal end of the explants in both the conduit and autograft groups, and axon density increased in the conduit group from the proximal to distal regions of the injury site. These phenomena, reduced axon area and increased axon density across an injury site, have been described for regenerating axons extending through both anastomosed transection injuries [59, 60] and conduits bridging segmental nerve injuries [61, 62]. The reduction in axon size has been associated with decreased maturation of regenerating axons [62]. Likewise, the conduit and autograft treated nerves exhibited a decrease in neurofilament and MBP myelin expression towards the distal end of the explant that was associated with a reciprocal increase in GAP43 expression [63]. Mature axons express more neurofilament whereas immature axons tend to express more GAP43 [60] further suggesting that the regenerating axons are more mature towards the proximal end and are progressively more immature towards the distal end of the conduits. Additionally, during regeneration, multiple axonal sprouts branch from axons proximal to the injury site leading to increased axon densities across nerve injuries; it is thought that these numerous axonal branches extend in search of the correct end organ and over prolonged repair times, extraneous axonal branches that fail to reach target organs subsequently atrophy [59, 60]. Consequently, in this study, the axons appear to still be in the process of regeneration and maturation across the injury site. The nerve repair process is not fully complete at the reported experimental end point, and recovery may continue such that, with time, the treated nerves may more closely histologically emulate healthy nerves.

Excitingly, our aligned DPSC conduits restored facial nerve function as well as the autograft, which is the clinical gold standard. In some animals, conduits greatly outperformed the autografts in whisker motion and CMAP amplitude, while the remaining functioned similarly to the autograft. Many factors could contribute to this range in conduit performance. Human DPSCs were utilized in these studies, and conduits were generated using cells isolated from different patients; potentially the biological variability across patient cells led to the noted variance in the regenerative capacity of the conduits. DPSCs isolated from certain individuals may have a genetic disposition to provide increased neurotrophic support. Alternatively, the total, heterogeneous population of DPSCs were utilized in these studies; certain cell populations within DPSCs may have greater repair properties in the nerve environment, and the fraction of this cell population could vary among patients. Overall, though, the results of this study confirm that DPSC scaffold-free nerve conduits stimulate nerve regeneration similar to the current standard of care, establishing a foundation for further investigating the use of these bioactive conduits in therapeutic applications. Uncovering the underlying factors that caused certain conduits to outperform autografts will allow for the optimization of the conduit design and consequently further improve nerve repair; this would ultimately enhance therapeutic outcomes in patients with facial nerve injuries.

Cells are driving the bioactivity of the scaffold-free aligned DPSC nerve conduits. It has been established that DPSCs endogenously express NTFs, and our previous studies substantiate that the processes of culturing DPSCs as cell sheets and inducing cell and ECM alignment does not affect global NTF expression levels [42]. Moreover, we confirmed that DPSC sheet secretome functionally induces neurite expression in neuronal cells *in vitro* [30]. Importantly, we found that these effects were reversed following targeted inhibition of NTF receptors further validating that the neuritogenic effects of DPSC sheet secretome are specifically due to NTFs. Although our focus thus far has been on assessing the neurotrophic behavior of the DPSC sheets, these stem/progenitor cells are likely involved in regulating numerous additional biological processes at the nerve injury site including modulating inflammation or inducing proliferation of native SCs [64].

In the current study, the DPSCs were not induced to differentiate. DPSCs were cultured in a standard growth medium supplemented with ascorbic acid to stimulate ECM maintenance enabling the formation of a robust cell sheet. The medium was also supplemented with FGF2, which we previously established increases the cellularity of the cell sheets and correspondingly the neurotrophic bioactivity [30]. We have found that inducing Schwann cell differentiation in aligned DPSC sheets increases NTF expression and the expression of Schwann cell basal lamina proteins yielding a unique biomaterial for conduit formation [65]. We elected in this study to first evaluate the regenerative effects of the undifferentiated DPSC conduits because our previous study showed that inducing Schwann cell differentiation reduced the cellularity and thickness of the aligned DPSC sheets. Therefore, additional fabrication techniques, like cell sheet stacking, will likely be required to form robust conduits using SC-induced DPSC sheets. Furthermore, Schwann cell differentiation involved a lengthy multi-step process, which may also reduce efficacy when generating conduits for therapeutic purposes. SC-differentiated conduits may regulate the nerve injury environment in a unique manner as those generated by undifferentiated DPSCs. Future studies evaluating how the differentiation state of the DPSCs impacts nerve repair would yield important new scientific insight and valuable information for therapeutic applications.

The aligned DPSC conduits exhibited relatively similar cross-sectional areas and mechanical properties as rat facial nerves. Nerve conduits must exhibit sufficient mechanical integrity to support the regenerating axons without being too stiff as to hinder regeneration or add extraneous strain to surrounding nerve structure. Furthermore, conduit stiffness has been shown to affect cell motility, neurite extension, and Schwann cell NTF expression *in vitro* [66]. Similarly, discrepancies in cross-sectional area between native nerve tissues and conduits can also result in impaired axon regeneration [67]. The relatively comparable geometry and stiffness of the between the aligned DPSC conduits and rat facial nerves indicates minimal mismatch between the implant and the injured nerve, further supporting their therapeutic potential. Moreover, these properties can be scaled up to match that of human facial nerves by stacking the cell sheets prior to conduit formation or fusing already formed conduits.

## 5. Conclusions

In this study, we demonstrated that our novel scaffold-free nerve conduits engineered from neurotrophic DPSCs and their endogenous aligned ECM are a viable alternative to current facial nerve therapies. By both enhancing and directing axon extension, these aligned DPSC conduits induced regeneration of myelinated nerve fibers across a critical sized segmental defect, resulting in functional recovery of whisker movement. The efficacy and safety of therapies using either autologous and allogenic DPSCs and their easy accessibility supports the likelihood of translating these scaffold-free DPSC conduits from bench to a clinical therapy, thus providing a therapeutic intervention that improves both functional outcomes of facial nerve repair and patient quality of life.

## Data Availability

The data that support the findings of this study are openly available at the following URL/DOI: https://doi.org/10.5281/zenodo.10594696. Data will be available from 30 January 2025.
